# Measuring recovery in participants with a schizophrenia spectrum disorder: validation of the Individual Recovery Outcomes Counter (I.ROC).

**DOI:** 10.1186/s12888-023-04763-3

**Published:** 2023-04-28

**Authors:** B. Esther Sportel, Hettie Aardema, Nynke Boonstra, Johannes Arends, Bridey Rudd, Margot J. Metz, Stynke Castelein, Gerdina H.M. Pijnenborg

**Affiliations:** 1 Department of Psychotic Disorders Assen, GGZ Drenthe Mental Health Institute, Dennenweg 9, 9404 LA Assen, The Netherlands; 2 Research Group Care and Innovation in Psychiatry, NHL Stenden University for Applied Sciences, Leeuwarden, The Netherlands; 3 Mental Health Foundation, Glasgow, Scotland; 4 GGz Breburg Mental Health Institute, Breda, The Netherlands; 5 Lentis Research, Lentis Psychiatric Institute, Groningen, The Netherlands; 6 Department of Clinical and Developmental Neuropsychology, Faculty of Behavioural and Social Sciences, University of Groningen, Groningen, The Netherlands

**Keywords:** Validation, Psychometric properties, Recovery, Schizophrenia spectrum disorder, Flexible assertive community treatment

## Abstract

**Background:**

To improve recovery in mental health, validated instruments are needed.

**Aims:**

This study evaluates psychometric properties of the Individual Recovery Outcomes Counter (I.ROC) in a Dutch population of participants with a schizophrenia spectrum disorder (SSD).

**Methods:**

326 participants completed the I.ROC at baseline (n = 326), six months (n = 155) and twelve months (n = 84) as part of a routine outcome assessment. Reliability, validity, sensitivity to change, and internal factor structure were examined.

**Results:**

Participants evaluated the I.ROC as comprehensive. Internal consistency of the I.ROC (α = 0.88) and test-retest reliability (r = .85, p < .001) are good. Negative moderate correlations with the total score of the PANSS (r=-.50, p < .001) and the HoNOS (r=-.52, p < .001) were found, and a small negative correlation with the FR tool (r=-.36, p < .001). Moderate positive correlation with the MANSA (r = .55, p < .001) and the RAS (r = .60, p < .001) were found. The mean total I.ROC scores increased significantly between time points (F(2,166) = 6.351, p < .005), although differences were small. Confirmatory factor analysis showed that fit indices for the one-, two-, and four-factor model are comparable.

**Conclusions:**

The I.ROC is a valid and reliable instrument, with sensitivity to change, to map recovery in participants with SSD.

## Introduction

Recovery has commonly come to be understood as living a rewarding and fulfilling life in the (ongoing) presence of a mental illness [1–4], and is now a central concept within current mental health care. The recovery movement, rooted in the psychiatric liberation, civil rights movement, and grass-roots activism of the 1970’s, advocated for the rights of people with mental illness to participate equally in society. First person accounts from people who have experienced mental illness and recovery combined with longitudinal research shining new light on the course and outcomes of mental illness [5], has enabled the reframing of the recovery concept. Consequently, over recent decades focus in treatment has moved from mainly symptom reduction (clinical recovery) to a more holistic model incorporating societal and personal aspects of recovery as well [4, 6–9].

Societal recovery is the extent to which someone is able to fulfil desired roles in his or her life [6], such as being a parent, child, neighbour, or employee. Personal recovery involves giving meaning to events from the past and incorporating mental illness as one aspect of a much broader personal identity [9]. According to Leamy et al. [10] key components of personal recovery are Connectedness, Hope and optimism, Identity, Meaning in life and Empowerment (CHIME). This CHIME framework has been recommended as a gold standard for measuring personal recovery [4, 11].

Recovery is known to be a unique process of an individual, and is a personal journey for each person [12]. Mental health care professionals can support recovery by gaining a deeper understanding of their client’s individual needs and wishes, including what is important to them in terms of recovery, and tracking change in these personal outcomes over time. In order to measure outcomes that are both personally and clinically relevant, an intervention’s effectiveness should be evaluated on all recovery dimensions; clinical recovery, societal recovery and personal recovery [8, 13, 14]. By gaining this insight, interventions can be personalised for each client, enabling a greater focus on enhancing recovery and quality of life. Recovery measurements can help enhance shared decision making, and monitoring recovery during treatment [15, 16].

### Measuring clients’ recovery

Standardised instruments are available that measure concepts closely related to clinical recovery (e.g. PANSS [17]), and societal recovery (MANSA, HoNOS [18, 19]), as well as a growing battery of personal recovery measures [20]. The need for an integrated view of recovery is widely shared, and several researchers are working to develop more integrated recovery measures, however, they are hardly validated or available yet [21]. The Individual Recovery Outcomes Counter [22] was developed for this purpose.

The I.ROC was developed in 2007 by the Scottish mental health charity Penumbra. Initially developed by a group of senior managers and mental health practitioners in collaboration with service users, the preliminary draft was improved following focus groups with service users and staff. Changes were approved during another round of focus group discussions with service users and by staff [23].

The I.ROC is divided into four domains forming the acronym HOPE (Home, Opportunity, People, and Empowerment) and contains twelve topics related to clinical (item 1,4,5), societal (item 2,3,6,7,8) and personal (item 9,10,11,12) recovery. Eight I.ROC-items (3, 6–12) cover personal recovery and some elements of societal recovery correspond to the five themes of the CHIME framework. The twelve items contain the following indicators: mental health, life skills, safety and comfort, physical health, exercise and activity, purpose and direction, personal network, social network, valuing myself, participation and control, self-management, and hope for the future. Unlike most recovery measures, the I.ROC was developed to initiate a dialogue on recovery. It can be used to help formulate personalized recovery goals and guides care in line with these goals [22]. Recovery can be seen as a journey as well as an outcome, by repeatedly administering the I.ROC, the recovery process becomes visible and treatment can be adjusted based on the results of the I.ROC. I.ROC results are visually presented in a spidergram showing individual areas of personal strength, unmet needs, and individual changes over time. This enables the service user and professional to work together on the recovery process [22]. Preliminary validation testing of the I.ROC (N = 170) took place in Scotland, with participants in the community receiving support from Penumbra. Participants’ most frequently self-reported mental illness diagnoses were common mental health problems such as depression and/or anxiety [24]. Results showed the I.ROC to have good internal consistency (α = 0.86). Comparative validity showed that the I.ROC scores are significantly positively correlated to scores of the Recovery Assessment Scale (RAS, r = .72, p < .001). In comparison to the BASIS-32 (Behavior and Symptom Identification Scale; [25] a significant negative correlation was found (r = -.60, p < .001). Initial exploratory factor analysis revealed two underlying factors, labelled as intrapersonal and interpersonal recovery [24], however, a later Rasch analysis [26] on a much larger sample implicates that the I.ROC represents a unidimensional construct. Rasch analysis is based on the item response theory, rather than classical test theory, and focuses on the fit between the actual score and the predicted score form the Rasch model [26]. Within the Netherlands, the I.ROC has been validated for a low-intensity community mental healthcare setting [27], showing psychometric properties comparable to previous studies and some evidence for sensitivity to change is found. They conclude that the I.ROC is a valid and reliable instrument to measure recovery in low-intensity community mental healthcare, but information about its use in people receiving high-intensity community care, diagnosed with a schizophrenia spectrum disorder, is lacking. The aim of this study is to examine the psychometric properties of the Dutch version of the I.ROC in a sample of participants with a schizophrenia spectrum disorder. In this study we compare the I.ROC with several frequently used measures of clinical, societal and personal recovery, and quality of life.

## Methods

### Translation of the I.ROC

The I.ROC was translated from English into Dutch by a group of researchers, practitioners, participants, and experts by experience. It was back-translated by NST Science (http://www.nstscience.nl/), an independent translation agency, and then presented to the developers for comments, resulting in some adjustments. Discussion points were presented to the research team and the translation agency, after which the final version was approved by the original authors. Translation guidelines as suggested by Van Widenfelt et al. [28] were followed.

### Participants and procedure

The study was carried out from June 2016 until December 2018 in Flexible Assertive Community Treatment (FACT) teams [29] across four outreach mental health care services; GGZ Drenthe Mental Health Institute, GGZ Friesland Mental Health Care Service, Lentis Psychiatric Institute and GGz Breburg Mental Health Institute. Inclusion criteria were: age 18 to 65, diagnosed with a schizophrenia spectrum disorder as established by a psychiatrist or psychologist, able to give written informed consent, sufficient mastery of the Dutch language, receiving care for at least one year.

To assess content validity, a sample size ≥ 7 is required [30]. Seven participants with a schizophrenia spectrum disorder were therefore invited to participate by their clinicians. For the evaluation of the other psychometric properties of the I.ROC, participants with a schizophrenia spectrum disorder, who were invited for annual routine outcome assessment [31], were asked to participate. Based on Clark and Watson [32], a sample size of ≥ 300 is required and the COSMIN criteria suggest at least 7 participants per question [30], both criteria are met in current study.

Eligible participants were informed about the study procedures, and then asked for written informed consent. The Medical Ethics Committee (METC) of the University Medical Centre Groningen concluded that assessment with the I.ROC falls beyond the scope of the Medical Research Involving Human Subjects Act (WMO) (2016-02-23, number M16.188934). The study has been approved by the local scientific committees of all four participating mental health care institutes.

As a first step in current validation, service users were asked about their opinions on the instrument. To prevent socially desirable answers two other recovery measures (RAS [20], and NHS [33]) were added. Participants were asked about relevance and comprehensiveness of the recovery measures. The order of the presented measures varied per participant.

In the second phase of validation testing, participants were assessed at baseline (t0), after six months (t1) and after twelve months (t2) with the I.ROC and a battery of additional questionnaires; PANSS [17], FR tool [21, 34], HoNOS [19], MANSA [18], and RAS [20] to assess validity and sensitivity to change. To assess test-retest reliability, participants completed the I.ROC twice, fourteen days apart with the same assessor under the same conditions (e.g. time of day, day of week). To ensure a robust test-retest protocol, participants needed to remain stable; this was monitored by their case manager. Data collection was carried out by trained research nurses, Bachelor of Nursing students and experts by experience.

### Measures

The *Individual Recovery Outcomes Counter* [24] comprises 12 items scored on a 6-point Likert scale from 1 (never) to 6 (all the time). Higher scores are reflective of greater progress towards personal recovery. For a more thorough description see above.

The *Positive and Negative Syndrome Scale* (PANSS [17]) is an instrument for typological and dimensional assessment of psychotic symptoms and is clinician-rated. The PANSS comprises 30 items scored on a 7-point Likert scale from 1 (absent) to 7 (extreme), with higher scores indicating more symptoms. The PANSS consists of three subscales; positive symptoms, negative symptoms, and general psychopathology. PANSS ratings are based on a semi-structured interview. Psychometric properties appear good; the internal consistency is acceptable (α = 0.79 [17]).

The *Functional Remission tool* (FR tool [21, 34]) is a 3-item instrument assessing societal recovery in people with a severe mental illness on three domains: living and self-care, work and study, and social contacts. The FR tool is a clinician-rated semi-structured interview with the patient or a significant other. Scores range from 0 to 2, with higher scores indicating less remission. Psychometric properties were evaluated; internal consistency is acceptable (α = 0.70 [21, 34]).

The *Health of the Nation Outcome Scales* (HoNOS [19]) is an instrument assessing mental and societal functioning of a client. The HoNOS is clinician-rated and consists of twelve items, scores range from 0 (no problem) to 4 (very severe problem). A review on the psychometric properties of the HoNOS showed an internal consistency ranging from 0.59 to 0.76 [35].

The *MANchester Short Assessment of quality of life* (MANSA [18]) is an instrument for assessing quality of life focusing on satisfaction with life as a whole and with different life domains including physical and mental health. This self-report questionnaire contains twelve items scored on a 7-point Likert scale. Scores range from 0 (very dissatisfied) to 6 (very satisfied). Psychometric properties of the MANSA have been tested and the internal consistency is acceptable (α = 0.74 [18] to good (α = 0.81 [36]).

The *Recovery Assessment Scale* (RAS [20]) is a self-report personal recovery questionnaire. The original version consists of 41 questions, but shorter versions are known. We used the RAS-24 to assess convergent validity of the I.ROC. Items are scored on a 5-point Likert scale, ranging from 1 (strongly disagree) to 5 (strongly agree). High scores indicate more recovery. A review of the psychometric qualities of the RAS [37] describes that the internal consistency in various studies is acceptable to excellent (α = 0.76- 0.97). The review mentioned significant positive correlations with the RAS and measures concerning quality of life, meaning of life, empowerment, self-esteem, sense of mattering, and hope.

### Analysis plan

To assess content validity, we conducted a qualitative pilot study using a semi-structured interview by an experienced interviewer. Interviews were recorded and transcribed verbatim and were analysed by two trained researchers using ATLAS.ti 8 Windows.

Internal consistency of the I.ROC and the two underlying factors (intrapersonal and interpersonal [24]) were calculated using Cronbach’s alpha, with α ≥ 0.70 as acceptable. Test-retest reliability of the I.ROC was analysed measuring the strength of the correlation and the concordance between the two I.ROC assessments fourteen days apart using the Pearson’s correlation coefficient and the Intraclass Correlation Coefficient (ICC; model two way random, type consistency [38, 39]). Values equal to or larger than 0.70 are considered acceptable. To calculate convergent validity, Pearson’s correlation coefficient was used. Coefficients from 0.10 to 0.39 are considered weak, 0.40–0.59 moderate and correlations of 0.60 or above as strong. We expected a moderate correlation between the I.ROC and the comparative measures since most of them measure only one or two recovery domains and are clinician-reported instead of self-reported.

Sensitivity to change over time was assessed by comparing the assessments at baseline (t0), six months (t1) and 12 months (t2) using one-way repeated-measures ANOVA as suggested by Stratford & Riddle [40]. The difference between I.ROC total scores at t0 and t1 was compared with the difference of the total score of the RAS and MANSA at t0 and t1 using the Pearson’s correlation coefficient to measure the strength of the correlation. Data was analysed with SPSS version 23 for Windows. We expected very little change between the time-points, since recovery on all three domains for people with schizophrenia spectrum disorder often takes years [14].

A confirmatory factor analysis for categorical data using multidimensional item response theory was conducted to examine whether the twelve questions can be divided into the two underlying dimensions as suggested by Monger et al. [24], or should be handled as one-factor as suggested by Dickens et al. [26]. We compared the two-factor model with the one-factor model, and with the original four-factor model, which is based on the HOPE model of the I.ROC. Fit indices were selected in order to test which model best represents the present dataset [41]: root-mean-squared error of approximation (RMSEA; a cut-off value close to 0.06), Tucker-Lewis index (TLI; cut-off value close to 0.90, higher is better) and comparative fit index (CFI; cut-off value close to 0.90, higher is better). Data for the CFA was analysed in R (version 3.6.0; www.r-project.org/), using the lavaan package for Structural Equation Modeling [42].

## Results

Seven participants were included to evaluate the content validity of the I.ROC, four women and three men. For the test-retest reliability 48 participants filled out the I.ROC twice two weeks apart (female: n = 32; age range: 23–63; mean age 47 (SD = 9.83)). In the evaluation of psychometric properties 326 participants were included for t0 (GGZ Drenthe Mental Health Institute (n = 74), GGZ Friesland Mental Health Care Service (n = 24), Lentis Psychiatric Institute (n = 59) and GGz Breburg Mental Health Institute (n = 171), of the participants 119 were female (36.3%), with age range from 24 to 65, and mean age 47 (SD = 10.66), 155 participants completed t1 (6 month follow-up), and 84 completed t2 (12 months follow-up). Reasons for drop out varied and included personnel changes, high workload of case managers, admission to clinical facilities or discharge of participants, no-show, and participants refraining from further participation. We compared completers and drop-outs on baseline for demographic variables and total scores of all instruments, only one significant difference was found, namely for the HoNOS (*t*(257) = 2.88, p = .04), with higher scores for drop-outs. A higher score on the HoNOS is indicative of more severe problems with functioning. Table 1 shows mean scores and standard deviations of all measures at baseline.

**Table 1:** Descriptive Statistics of I.ROC, RAS, PANSS, MANSA, HoNOS and FR tool at baseline

Instrument	n	Range	Mean (SD)
I.ROC	326	17–72	48.81 (10.02)
RAS	287	28–120	87.08 (12.10)
PANSS	101	30–73	45.06 (9.58)
MANSA	265	20–84	51.11 (11.77)
HoNOS	259	0–30	8.61 (5.62)
FR tool	237	0–12	2.14 (1.74)

*I.ROC = Individual Recovery Outcomes Counter; RAS = Recovery Assessment Scale; PANSS = Positive and Negative Syndrome Scale; MANSA = MANchester Short Assessment of quality of life; HoNOS = Health of the Nation Outcome Scale; FR tool = Functional Remission tool*

### Content validity

All seven interviewed participants were positive about the user-friendliness of the I.ROC. Participants reported that the questionnaire is short, and questions were easy to understand. The items were considered recovery-oriented and relevant to participants’ own experience. When asked, all participants confirmed that the I.ROC helps to start a dialogue about their recovery process. Most participants (n = 6) mentioned that I.ROC focuses on strengths and not just on weaknesses or complaints. Six participants stated that the visual representation of their answers in a spidergram provides insight into their recovery process and helps formulate personal goals and wishes. All participants agreed that the I.ROC should be used as a model to facilitate a conversation rather than a quick assessment.

### Reliability

#### Internal consistency

The I.ROC showed good internal consistency as assessed with Cronbach’s alpha (α = 0.88). Internal consistency of the two underlying factors is; α = 0.85 (intrapersonal) and α = 0.71 (interpersonal).

#### Test-retest reliability

Test-retest reliability was good (n = 48; r = .85, p < .001). The single measure intraclass correlation coefficient was 0.85 with a 95% confidence interval from 0.75 to 0.92 (F(47,47) = 12.60, p < .001).

### Convergent validity

Small to moderate negative correlations were found between the I.ROC and the PANSS total score (r = − .50, p < .001) and all three subscales (PANSS positive symptoms: r = − .37, p < .001, PANSS negative symptoms: r = − .28, p = .002, and PANSS general psychopathology: r = − .46, p < .001), the HoNOS (r = − .52, p < .001) and the functional remission tool (r = − .36, p < .001). There were moderate positive correlations with the MANSA (r = .55, p < .001) and the RAS (r = .60, p < .001). When testing correlations between the total scores of the PANSS, FR tool, HoNOS, MANSA, and RAS, and the PANSS subscales with the specific questionnaire-relevant I.ROC items based on the relevant recovery dimensions, results were comparable or slightly lower (see Table 2).

**Table 2:** Convergent validity of I.ROC based on the correlations and confidence intervals of I.ROC subscores with the comparative scales

Comparative instruments	I.ROC clinical		I.ROC clinical and societal		I.ROC societal		I.ROC personal	
	r	CI	r	CI	r	CI	r	CI
PANSS total score	− 0.32	0.13,-0.49	NA	-	NA	-	NA	-
PANSS positive	− 0.24	− 0.38,-0.06	NA		NA		NA	
PANSS negative	− 0.12	− 0.27,0.06	NA		NA		NA	
PANSS genpsy	− 0.30	− 0.44,-0.11	NA		NA		NA	
HoNOS total score	NA	-	− 0.51	0.41-0.60	NA	-	NA	-
MANSA total score	NA	-	0.55	0.46-0.63	NA	-	NA	-
FR tool total score	NA	-	NA	-	− 0.37	0.26-0.48	NA	-
RAS total score	NA	-	NA	-	NA	-	0.60	0.52-0.67

*I.ROC = Individual Recovery Outcomes Counter; RAS = Recovery Assessment Scale; PANSS = Positive and Negative Syndrome Scale (pos = positive, neg = negative, genpsy = general psychopathology); MANSA = MANchester Short Assessment of quality of life; HoNOS = Health of the Nation Outcome Scale; FR tool = Functional Remission tool*

*I.ROC clinical recovery related items: 1 + 4 + 5, I.ROC societal recovery related items: 2 + 3 + 6 + 7 + 8, I.ROC personal recovery related items 9 + 10 + 11 + 12*

### Sensitivity to change over time

A one-way repeated measures ANOVA (n = 84) determined that the mean total I.ROC score increased significantly between time points (F(2, 166) = 6.35, p < .05). The variance of the data points was in line with the assumption of sphericity, χ(2) = 0.99, p = .64. Post hoc tests using the Bonferroni correction revealed a significant increase in scores from t0 to t1 (48.88 vs. 51.10, p < .05), and from t0 to t2 (48.88 vs. 51.40, p < .05). The difference between the mean total I.ROC scores from t1 to t2 (51.10 vs. 51.38) was not statistically significant (p > .05). Small within participants changes between time-points were detected as expected in the mean total scores of I.ROC, RAS and MANSA (Table 3). Pearson’s correlations revealed a small positive correlation between the change in I.ROC totals from t0 to t1 and the change in RAS (r = .23, p < .01) and MANSA (r = .27, p < .01) (effect sizes ranging from 0.04 to 0.08).

**Table 3:** Mean total scores at baseline (t0), after six months (t1) and after twelve months (t2) in order to assess the sensitivity to change over time

Instrument	Mean total score T0	Mean total score T1	Mean total score T2
I.ROC (n = 84)	48.88	51.10	51.38
RAS (n = 66)	84.89	88.12	87.89
MANSA (n = 49)	51.33	53.90	53.90

*I.ROC = Individual Recovery Outcomes Counter; RAS = Recovery Assessment Scale; MANSA = MANchester Short Assessment of quality of life*

### Confirmatory factor analysis

According to the fit indices, the two-factor model (inter- and intrapersonal recovery) showed a similar fit as the one-factor and four-factor model (HOPE-model) (Table 4).

**Table 4:** Comparison of the three models through CFA to test for the best fit

Indices	CFI	TLI	RMSEA
One-factor model	0.912	0.892	0.088
Two-factor model	0.915	0.894	0.087
Four-factor model	0.913	0.881	0.092

*CFI = comparative fit index; TLI = Tucker-Lewis index; RMSEA = root-mean-squared-error of approximation*

## Discussion

An integrated instrument to measure recovery is of importance, especially for people with a serious mental illness, given the impact of their condition on almost all aspects of life. The I.ROC could be that instrument, therefore we looked into its psychometric properties in a sample of people with schizophrenia spectrum disorder. Results show that participants found the I.ROC comprehensive. Internal consistency and test-retest reliability are good, and convergent and divergent validity are as predicted, making it a useful instrument for people with schizophrenia spectrum disorder.

Interviews showed that service-users are enthusiastic about the I.ROC. They appreciated the instrument as an important facilitator to start a dialogue about their own personal recovery process. The spidergram provided them with an overview of the progress they had made towards recovery, and stimulated the formulation of personal goals and wishes for the future, enhancing a sense of ownership within patients. Patients also report that the IROC encourages dialogue about their recovery process and focuses on strengths and not just on complaints.

The test-retest reliability of the I.ROC is good, as is the internal consistency. Confirmatory factor analysis showed comparable fits for the tested models. Correlations with all measures included in the assessment were in the predicted direction and were as strong (moderate) [43] or stronger than hypothesised, supporting the convergent validity of the I.ROC with measures of clinical, societal and personal recovery. As expected, the I.ROC correlated negatively with clinical symptoms (PANSS) and mental and societal functioning (HoNOS), meaning that higher I.ROC scores (i.e. more recovery), are correlated with less symptoms and more societal and clinical recovery. Although the correlation on societal recovery as assessed with the HONOS was moderate, the correlation with functional remission was weak. The FR tool consists only of three questions and is clinician rated. Unlike the I.ROC, the FR tool assesses both the client’s skills (capacity) and actual behaviour (performance) [21, 34]. Also in line with our hypotheses on convergent validity, positive correlations were found between I.ROC, MANSA and RAS, meaning higher I.ROC scores are correlated with more societal and personal recovery. The correlation with the clinical symptoms (PANSS) and the three I.ROC clinical recovery items was weaker than expected, suggesting the I.ROC does not specifically reflect clinical recovery. This might be due to the small (n = 3) number of items included in this domain. On the other hand it should be noted that the I.ROC is a self-report measure, filled out by the participant, while the PANSS is clinician rated. Recovery is a personal process, and since the I.ROC focuses on personal experience, our findings could be an expression of subjective experience. This also yielded for the other subdomains after dividing the I.ROC items into clinical, societal and personal recovery.

Changes in I.ROC scores over time are in line with changes as measured with personal recovery measure RAS, of which some sensitivity to measure change is assumed [37], and in line with two other studies on the I.ROC showing some evidence of sensitivity to change [27, 44]. Between time points the differences of the mean total I.ROC, RAS, and MANSA scores are comparable and all in the same direction, but very small. It is known that change over time in this population is small [14], given the short period between our assessments, we might have missed a larger change that occurred over a longer period of time. It often takes years to fulfil desired rolls and rebuild identity [45].

In the confirmatory factor analysis the two-factor model showed a comparable fit to the one- and four-factor model. Dickens et al. [26] concluded that, given the high correlation between the factors, the I.ROC measures a unidimensional construct. The two factors identified within the initial psychometric study may however provide insight into the resilience of an individual patient [24], and can therefore be useful in treatment. All results are comparable to those of the Dutch I.ROC validation study in a low-intensity community mental healthcare setting [27], indicating I.ROC can be used in multiple settings and within multiple patient groups.

### Limitations

A substantial number of participants dropped out at t1 and t2, in schizophrenia research drop-out is considered a problem within psychosocial treatment as well as medical trials [46–48]. Drop-out might not have been totally at random, since we found a difference at baseline on the HoNOS. Participants with higher scores, and thus more functional problems, dropped out more frequently, indicating that continued participation could be too big a burden on them. Unfortunately, exact numbers for drop-out reasons were not recorded, since the data was collected for clinical purposes as part of the yearly routine outcome assessment.

The drop-out rate only affected analysis of sensitivity to change. Our other analysis only included baseline data, resulting in less power to detect change. The small sensitivity to change is comparable to that of the Recovery Assessment Scale, and within the current population we didn’t expect a large change over time. Another limitation is the fact that participants have been followed for a maximum of twelve months; this could have been too short a time over which to detect changes in societal and personal recovery.

Sensitivity to change should be more thoroughly investigated in further research. A larger sample-size and a more longitudinal approach in which participants who relapse or recover must remain included, could help to better detect changes in recovery. To implement the use of the I.ROC more broadly, the psychometric properties should be evaluated in other patient groups in mental health care.

After our data collection began it was shown that the 6-point scale can problematic and a 4-point scale of the I.ROC is advised [26], future research on the 4-point scale should give more clarity on the subject, particularly in relation to change over time.

Furthermore, we need to consider that especially personal recovery is an especially complex and subjective concept, it is a unique and personal process, a journey and not an outcome [12] with fixed cut-off values. This makes quantification complicated.

### Conclusions and implications for practice

Based on the results of this validation study we may conclude that the I.ROC is a reliable and valid instrument and includes all recovery domains (clinical, societal and personal) and can be used to measure recovery in people with a schizophrenia spectrum disorder. Participants who participated in this study were positive about the I.ROC. They appreciate the I.ROC because it is self-reported; the 12 items are relevant to their own recovery process and well-being; it stimulates the dialogue about their own recovery process. The I.ROC focuses on strengths and not so much on weaknesses or problems and it is easy to use and short. The I.ROC can be useful in following processes in recovery oriented interventions or activities, giving a boost to the implementation of these interventions. A structural implementation of the I.ROC in treatment evaluations could help keeping a recovery oriented focus in treatment.

## Data Availability

The datasets generated and/or analysed during the current study are not publicly available, since participants gave written informed consent for their data to be used in analysis, but no written informed consent for their data to be made publicly available. Upon reasonable request data are available from the corresponding author.
